# Clinical, Immunological and Treatment-Related Factors Associated with Normalised CD4+/CD8+ T-Cell Ratio: Effect of Naïve and Memory T-Cell Subsets

**DOI:** 10.1371/journal.pone.0097011

**Published:** 2014-05-09

**Authors:** Willard Tinago, Elizabeth Coghlan, Alan Macken, Julie McAndrews, Brenda Doak, Charlotte Prior-Fuller, John S. Lambert, Gerard J. Sheehan, Patrick W. G. Mallon, on behalf of the Mater Immunology Study Group

**Affiliations:** 1 HIV Molecular Research Group, School of Medicine and Medical Science, University College Dublin, Dublin, Ireland; 2 Department of Community Medicine, University of Zimbabwe, Harare, Zimbabwe; 3 Department of Immunology, Mater Misericordiae University Hospital, Dublin, Ireland; 4 Department of Infectious Diseases, Mater Misericordiae University Hospital, Dublin, Ireland; University of Nebraska Medical center, United States of America

## Abstract

**Background:**

Although effective antiretroviral therapy(ART) increases CD4+ T-cell count, responses to ART vary considerably and only a minority of patients normalise their CD4+/CD8+ ratio. Although retention of naïve CD4+ T-cells is thought to predict better immune responses, relationships between CD4+ and CD8+ T-cell subsets and CD4+/CD8+ ratio have not been well described.

**Methods:**

A cross-sectional study in a cohort of ambulatory HIV+ patients. We used flow cytometry on fresh blood to determine expanded CD4+ and CD8+ T-cell subsets; CD45RO+CD62L+(central memory), CD45RO+CD62L-(effector memory) and CD45RO-CD62L+(naïve) alongside routine T-cell subsets(absolute, percentage CD4+ and CD8+ counts), HIVRNA and collected demographic and treatment data. Relationship between CD4+/CD8+ T-cell ratio and expanded T-cell subsets was determined using linear regression analysis. Results are median[IQR] and regression coefficients unless stated.

**Results:**

We recruited 190 subjects, age 42(36–48) years, 65% male, 65.3% Caucasian, 91% on ART(52.6% on protease inhibitors), 78.4% with HIVRNA<40cps/ml and median ART duration 6.8(2.6–10.2) years. Nadir and current CD4+ counts were 200(112–309) and 465(335–607) cells/mm^3^ respectively. Median CD4+/CD8+ ratio was 0.6(0.4–1.0), with 26.3% of subjects achieving CD4+/CD8+ ratio>1. Of the expanded CD4+ T-cell subsets, 27.3(18.0–38.3)% were naïve, 36.8(29.0–40.0)% central memory and 27.4(20.0–38.5)% effector memory. Of the CD8+ T-cells subsets, 16.5(10.2–25.5)% were naïve, 19.9(12.7–26.6)% central memory and 41.0(31.8–52.5)% effector memory. In the multivariable adjusted analysis, total cumulative-ART exposure(+0.15,p = 0.007), higher nadir CD4+ count(+0.011,p<0.001) and higher %CD8+ naive T-cells(+0.0085,p<0.001) were associated with higher CD4+/CD8+ ratio, higher absolute CD8+ T-cell(-0.0044,p<0.001) and higher %CD4+ effector memory T-cells(-0.004,p = 0.0036) were associated with lower CD4+/CD8+ ratio. Those with CD4+/CD8+ ratio>1 had significantly higher median %CD8+ naive T-cells; 25.4(14.0–36.0)% versus 14.4(9.4–21.6)%, p<0.0001, but significantly lower absolute CD8+ count; 464(384.5–567) versus 765(603–1084) cells/mm^3^, p<0.001.

**Conclusions:**

Study suggests important role for naïve CD8+ T-cell populations in normalisation of the immune response to HIV-infection. How these findings relate to persistent immune activation on ART requires further study.

## Introduction

Human immunodeficiency virus infection is characterized by CD4+ T-cell depletion, CD8+ T-cell expansion and chronic immune activation that leads to immune dysfunction [1]. The mechanism of CD4^+^ T-cell depletion differs in the acute and chronic phases [2].

The dynamics of CD4+ and CD8+ T-cells are altered in many ways during HIV infection, with both showing evidence of early increased proliferation and subsequent preferential loss of the naive subset as untreated infection progresses. Infection with HIV-1 is known to induce an early decline in the number of naive CD4+, naive CD8+ and memory CD4+ T cells [3], [4], [5], [6]. In contrast, the memory and activated CD8+ T-cell compartments expand initially. The overall result is depletion of the CD4+ T-cell pool and expansion of the CD8+ T-cell pool. Only shortly preceding progression to AIDS does the numbers of these latter cell types fall [7], [8].

CD4+ T-cell loss is associated with increased CD8+ T-cell activation and increased memory CD8+ T-cells [9], which are predictive of HIV disease progression and death [10]. ART helps to restore circulating T-cells by decreasing T-cell turnover and redistributing T-cells [11], [12]. However, inter-individual responses to HAART vary considerably and HIV-specific CD4+ T-cell responses are rarely recovered,[13] with normalisation of CD4+/CD8+ T-cell ratio occurring in only a minority of cases [14].

Failure to normalize the CD4+/CD8+ T-cell ratio despite peripheral CD4+ T-cell count restoration is a common observation in clinical practice; few studies have addressed the biological or the clinical significance of this phenomenon [15], despite evidence showing CD4+/CD8+ T-cell ratio to independently predict immune restoration [16]. Although retention of naïve CD4+ T-cells is thought to predict a better immune response, relationships between subsets of CD4+ and CD8+ T-cells and CD4+/CD8+ T-cell ratio have not been well described. This study aims to explore the relationship between CD4+/CD8+ T-cell ratio and naïve and memory CD4+ and CD8+ T-cells.

## Methods

### Study design, subjects and recruitment

We conducted a cross-sectional study on 190 ambulatory HIV-infected patients attending the Mater Misericordiae University Hospital (MMUH) infectious diseases outpatient clinic. Consecutive HIV infected patients were enrolled into the study during clinic visit, if they were aged≥18 years, able to provide written informed consent and undergo regular blood testing at routine clinic visits.

Subjects were enrolled into the study as part of a prospective cohort study to assess changes in CD4+, CD8+ T-lymphocytes subsets and CD4+/CD8+ T-cell ratio. We conducted the cross-sectional analysis using data from the subjects' point of entry into the prospective cohort study. In the prospective cohort study, patients were followed for a median 34 (13-57) weeks. The study was approved by the Mater Misericordiae University Hospital and Mater Private Hospital Research Ethics Committee. All patients provided written informed consent.

### T-lymphocyte subsets

Determination of circulating T-cells was carried out using the Becton Dickinson SPA11 processor (BD Biosciences, San Jose, California). Fresh EDTA samples were stained using fluorochrome labelled monoclonal antibodies from Becton Dickinson Biosciences (BD UK Limited, Oxford) in a lyse no wash method [17], [18]. The samples were processed in BD trucount tubes to allow absolute cell numbers of each cell population to be calculated. Samples were gated using CD3+, CD4+ and CD8+ antibodies and CD62L+ was used as a marker of T-cell memory and CD45RO+ as a marker of T-cell maturity (Figure S1). CD4+ and CD8+ T-cells subsets were defined using the following co-expression of markers: naïve cells (CD45RO-, CD62L+), central memory (CD45RO+, CD62L+), effector memory (CD45RO+, CD62L-) and revertant T-cells (CD45RO-, CD62L-)[19], [20]. Resulting quadrant gates were analysed post-processing using Becton Dickinson DiVa 6.1.2 software (BD Biosciences, San Jose, California). Laboratory specimen analyses were performed in the Department of Immunology at the Mater Misericordiae University Hospital.

### Study variables

The study outcome was CD4+/CD8+ T-cell ratio. Data on clinical and baseline demographic variables including age, gender, transmission risk, hepatitis co-infection, cumulative exposure to ART (total cumulative exposure and cumulative exposure to individual ART drugs and drug classes), nadir CD4+ T-cell count, current and baseline absolute CD8+ and CD4+ T-cell counts, baseline percentage CD4+ and CD8+ T-cell subsets and HIV RNA measured by Amplicor HIV-1 Monitor (Roche Molecular Systems, Branchburg, New Jersey, USA) were collected.

### Statistical analysis

Subjects continuous demographic and clinical outcome data were summarized as median (IQR) and categorical data were summarized as absolute numbers and percentages. Linear relationship between CD4+/CD8+ T-cell ratio and expanded T-cell subsets was assessed using scatter plots. Due to the skewed distribution of %CD8+ naïve and %CD8+ T-cell, differences in T-cell subsets between those who achieved a CD4+/CD8+ T-cell ratio≥1 and those who did not were compared using the Mann-Whitney U test. Multivariable linear regression analysis was used to examine the relationship between CD4+/CD8+ T-cell ratio and expanded T-cell subsets. We performed a multiple linear regression analyses in two stages. In the first stage, control variables age, gender, ethnicity, Hepatitis C and current HIV RNA were entered into the regression. In the second stage, covariates with p<0.10 in the univariate analysis were added (these included; treatment exposure variable, nadir CD4+ T-cell count, CD4+, CD8+ T-cells and their subsets) to the adjusted model and backward elimination used on the stage two covariates. The final model retained stage two variable if they had a p<0.05. When covariates were considered to be collinear (eg %CD4+ naive and %CD4 effector), the variable with the strongest association to the CD4+/CD8+ T-cell ratio was included in the multivariable model. Multicollinearity between independent variables in the final was assessed using the variance inflation factor (VIF). Linear regression assumptions were checked by exploring regession residuals. We conducted sensitivity analyses to examine whether similar effects are maintained when we consider only a subset of subjects with HIV RNA≤40 copies/ml [21]. Results of linear regression analyses were presented as regression coefficients with 95% confidence interval and p-values.

In a secondary analysis, we explored which of either CD4+/CD8+ T-cell ratio, CD4+ or CD8+ T-cell is better in informing on expanded T-cell subsets. For each expanded T-cell subset, three multivariable linear regression models with either CD4/+CD8+ T-cell ratio, CD4+ or CD8+ T-cell count as independent variable were fitted, adjusted for potential confounders. The Bayesian Information Criterion (BIC) and Alkake Information Criterion (AIC) were used to assess which of models with CD4/+CD8+ T-cell ratio, CD4+ T-cell or CD8+ T-cell count best explained an expanded T-cell subset. Analyses were performed using STATA 12.1 (College Station, Texas).

## Results

Between May 2011 and February 2013 we recruited 190 subjects. The group studied broadly represents the HIV epidemic in Europe, with both genders and all major ethnicities and HIV transmission groups represented. Subject demographic and treatment data are presented in table 1. The majority (91%) of subjects were on ART, with 91 (52.6%) on ritonavir-boosted protease inhibitors (PI/r) and, 72 (42.2%) on NNRTI containing ART regimens. Most (78.4%) subjects had suppressed HIV RNA (<40 cps/ml) and CD4+ T-cell recovery (465 (335, 607) cells/mm^3^). The median time since ART initiation and cumulative exposure to ART was 6.8 (2.6–10.2) years and 5.4 (2.2–9.5) years respectively. The median CD4+/CD8+ T-cell ratio was 0.6 (0.4–1.0) with 26.3% of the subjects achieving a CD4+/CD8+ T-cell ratio≥1.

**Table 1:** Subject demographic and treatment characteristics.

Characteristic	N = 190
Age (yrs)	42.1 (36.2–47.9)
Gender n (%)	
Female	67 (35.3%)
Male	123 (64.7%)
Ethnicity n (%)	
Caucasian	124 (65.3%)
African	55 (29.0%)
Other	11 (5.7%)
HIV transmission risk group n (%)	
Heterosexual	70 (36.8%)
IDU	57 (30.0%)
Homosexual	50 (26.3%)
Needle stick/Unknown	13 (6.9%)
Hepatitis C Ab n (%)	
Positive	60 (31.6%)
Negative	130 (68.4%)
Hepatitis B SAg n(%)	
Positive	6 (3.2%)
Negative	184 (96.8%)
Time since HIV diagnosis (years)	9.0 (5–13)
Nadir CD4+ T-cell count (cells/mm^3^)	200 (112–309)
Current CD4+ T-cell count (cells/mm^3^)	464.5 (334.5–607)
HIV RNA (copies/ml) n(%)	
≤40	149 (78.4%)
>40	41 (21.6%)
ART exposure n (%)	
Naïve	17 (8.9%)
Experienced	173 (91.1%)
Current PI use (n(%))	
lopinavir/r	6 (3.2%)
atazanavir/r	56 (29.5%)
darunavir/r	29 (15.3%)
Current NNRTI use (n(%))	
Efavirenz	56 (29.5%)
Nevirapine	12 (6.3%)
Rilpivirine	2 (1.1%)
Etravirine	2 (1.1%)
Current N(t)RTI use (n(%))	
tenofovir DS	137 (72.1%)
Emtricitabine	132 (69.5%)
Abacavir	29 (15.3%)
Lamuvidine	31 (16.3%)
Zidovudine	3 (1.6%)
Current INT use (n (%))	
Raltegravir	5 (2.6%)
Time since ART initiation (years)	6.8 (2.6–10.2)
Total cumulative ART exposure (years)	5.4 (2.2–9.5)
Total cumulative PI exposure (years)	4.4 (2.0–7.2)
Total cumulative NRTI exposure (years)	5.5 (2.2–9.5)
Total cumulative NNRTI exposure (years)	3.0 (1.0–6.7)
Total cumulative INT exposure (years)	0.9 (0.4–1.6)

Data are median (IQR) unless otherwise stated, IDU-intravenous drug use, Ab-antibody, SAg-surface antigen,PI-protease inhibitor, N(t)RTI-nucleoside (-tide) reverse transcriptase, NNRTI-non- nucleoside reverse transcriptase inhibitor, INT-integrase inhibitor.

Of the expanded CD4+ T-cell subsets, central memory (CD62L+CD45RO+) cells were the most represented subset followed by the effector memory cells (CD62L-CD45RO+) and naïve cells (CD62L+CD45RO-) respectively (table 2). In contrast, of the CD8+ T-cells subsets, effector memory cells were the most represented subset followed by central memory cells and naïve cells respectively.

**Table 2:** T-cell subsets.

Characteristic	N = 190
Current CD4+ T-cell count (cells/mm^3^)	464.5 (334.5–607)
%CD4+ T-cells	36.5 (26.9–45.8)
Absolute CD8+ cell count (cells/mm^3^)	681 (471.5–966)
%CD8+ T-cells	56.4 (45.5–64.8)
CD4+/CD8+ T-cell ratio	0.6 (0.4–1.0)
CD4+/CD8+ T-cell ratio≥1	50 (26.3 %)
%CD4+ naïve (CD45RO-CD62L+)	27.3 (18.0–38.3)
%CD4+ central memory (CD45RO+CD62L+)	36.8 (29–40)
%CD4 effector memory (CD45RO+CD62L-)	27.4 (20.0–38.5)
%CD8+ naïve (CD45RO-CD62L+)	16.5 (10.2–25.5)
%CD8+ central memory (CD45RO+CD62L+)	19.9 (12.7–26.6)
%CD8+ effector memory (CD45RO+CD62L-)	41.0 (31.8–52.5)

Data are median (IQR) unless otherwise stated.

### Association of CD4+/CD8+ T-cell ratio with expanded T-cell subsets and treatment-related variables

In the univariate analysis (table 3), in a model including all patients, being Hepatitis C antibody positive, IDU transmission risk group, higher absolute CD8+ T-cell count, higher %CD8+ T-cells, higher %CD8+ effector memory T-cells and higher %CD4+ effector memory T-cells were all associated with a lower CD4+/CD8+ T-cell ratio. Current HIV RNA<40copies/ml, cumulative exposure years to ART, cumulative exposure years to NRTI, cumulative exposure years to PI, time since ART initiation, higher nadir CD4+ T-cell count per 10 cell increase, higher current absolute CD4+ T-cell count per 10 cell increase, higher %CD4+ T-cells, higher %CD4+ naïve T-cells and higher %CD8+ naïve T-cells were all significantly associated with a higher CD4+/CD8+ T-cell ratio. In a univariate model which included only subjects on ART with suppressed HIV RNA (<40 cp/ml), similar associations were observed between the above covariates and CD4+/CD8+ T-cell ratio.

**Table 3:** Univariate linear regression analysis for factors associated with CD4+/CD8+ T-cell ratio.

	All patients (N = 190)			Patients on ART with HIV RNA<40 cp/ml (N = 149)		
	Coefficient	95% Confidence Interval	P-value	Coefficient	95% Confidence Interval	P-value
Age (per 10 year increase)	+0.049	−0.017, 0.12	0.15	−0.018	−0.11, 0.070	0.68
Gender:Female	+0.054	−0.08, 0.18	0.42	+0.095	−0.063, 0.25	0.24
Ethnicity:Caucasian	+0.033	−0.16,0.10	0.62	+0.083	−0.24, 0.075	0.30
HIV transmision risk:IDU	−0.15	−0.29, −0.018	0.03	−0.22	−0.38, −0.063	0.006
Hepatitis C Ab:Positive	−0.16	−0.29, −0.03	0.02	−0.23	−0.39, −0.076	0.004
Hepatitis B sAg:Positive	−0.048	−0.40, 0.31	0.79	−0.081	−0.48, 0.32	0.69
Time since HIV diagnosis (years)	+0.009	−0.0007, 0.02	0.07	+0.0019	−0.0091, 0.013	0.74
HIV RNA<40 cps/ml	+0.32	0.18, 0.46	<0.001	-	-	-
Time since ART initiation(years)	+0.024	0.010, 0.04	0.001	+0.019	0.0038, 0.035	0.015
Total cumulative ART exposure (years)	+0.027	0.015, 0.04	<0.001	+0.020	0.0043, 0.036	0.013
Cumulative exposure to PI (years)	+0.025	0.008, 0.041	0.004	+0.028	0.0022, 0.054	0.034
Cumulative exposure to NRTI (years)	+0.027	0.014, 0.04	<0.001	+0.020	0.0042, 0.036	0.014
Cumulative exposure to NNRTI (years)	+0.021	−0.002, 0.04	0.08	+0.016	−0.0072, 0.040	0.17
Nadir CD4+ T-cell count (per 10 cells/mm^3^)	+0.0066	0.030, 0.11	0.001	+0.014	0.0093, 0.020	<0.001
Absolute CD4+ T-cell count (per 10 cells/mm^3^)	+0.011	0.09, 0.13	<0.001	+0.011	0.0084, 0.013	<0.001
%CD4+ cells	+0.031	0.030, 0.03	<0.001	+0.033	0.031, 0.035	<0.001
Absolute CD8+ T-cell count (per 10 cells/mm^3^)	−0.0051	−0.06, −0.04	<0.001	−0.0061	−0.0081, −0.0040	<0.001
%CD8+ T-cells	−0.032	−0.03, −0.030	<0.001	−0.033	−0.036, −0.031	<0.001
%CD4+ naïve (CD45RO-CD62L+)	+0.0098	0.006, 0.014	<0.001	+0.011	0.0061,0.016	<0.001
%CD4+ central memory (CD45RO+CD62L+)	+0.0029	−0.002, 0.008	0.29	+0.00015	−0.0067, 0.0070	0.97
%CD4+ effector memory (CD45RO+CD62L-)	−0.013	−0.02, −0.008	<0.001	−0.013	−0.018, −0.0075	<0.001
%CD8+ naïve (CD45RO-CD62L+)	+0.013	0.008, 0.02	<0.001	+0.011	0.0046, 0.017	0.001
%CD8+ central memory (CD45RO+CD62L+)	+0.0059	−0.0002, 0.012	0.06	+0.00087	−0.0069, 0.0086	0.83
%CD8+ effector memory (CD45RO+CD62L-)	−0.0085	−0.013, −0.004	<0.001	−0.0064	−0.012, −0.00080	0.025

IDU-Intravenous drug use, Ab-antibody, sAg-surface antigen, PI-protease inhibitor, N(t)RTI-nucleoside (-tide) reverse transcriptase, NNRTI-non-nucleoside reverse transcriptase inhibitor.

NB: Coefficients of %CD4+, %CD8+ T-cells and their subsets are per 1% increase.

On multivariable analysis (table 4), adjusted for age, gender, ethnicity, Hepatitis C antibody status and current HIV RNA, in a linear regression model including all cohort subjects, cumulative exposure years to ART, higher nadir CD4+ T-cell count, and higher %CD8+ naive T-cells remained independently associated with a higher CD4+/CD8+ T-cell ratio. In the same adjusted model, higher absolute CD8+ T-cell count and higher %CD4+ effector memory T-cells remained independently associated with lower CD4+/CD8+ T-cell ratio.

**Table 4:** Adjusted multivariate linear regression analysis for factors associated with CD4+/CD8+ T-cell ratio.

	†All patients (N = 190)			¥Patients on ART with HIV RNA<40 cp/ml (N = 149)		
	Coefficient	95% Confidence Interval	P-value	Coefficient	95% Confidence Interval	P-value
Total cumulative ART exposure (years)	+0.014	0.0042, 0.025	0.006	+0.014	0.0022, 0.026	0.021
Nadir CD4+ T-cell count (per 10 cells/mm^3^ increase)	+0.011	0.0081, 0.014	<0.001	+0.013	0.0084, 0.017	<0.001
Absolute CD8+ T-cell count (per 10 cells/mm^3^ increase)	−0.0044	−0.0056, −0.0033	<0.001	−0.0052	−0.0068, −0.0036	<0.001
%CD4+ effector memory (CD45RO+CD62L-)	−0.0036	−0.0074, 0.00006	0.054	−0.0057	−0.010, −0.0012	0.014
%CD8+ naive (CD45RO-CD62L+)	+0.0088	0.0044, 0.013	<0.001	+0.0080	0.0028, 0.013	0.003

† Adjusted for age, gender, ethnicity, Hepatitis C status, and HIV RNA.

¥ Adjusted for age, gender, ethnicity, Hepatitis C status.

NB: Coefficients of %CD4+, %CD8+ T-cells and their subsets are per 1% increase.

To assesses the sensitivity of our analysis, we also fitted a multivariate model including only subjects on ART with suppressed HIV RNA (<40 cp/ml), adjusted for age, gender, ethnicity and Hepatitis C antibody status, similar direction of association was also observed between covariates; total cumulative exposure to ART, nadir CD4+ T-cell count, absolute CD8+ T-cell count, %CD4+ effector memory, %CD8+ naive T-cells with CD4+/CD8+ T-cell ratio(table 4).

Compared to subjects who had a CD4+/CD8+ T-cell ratio<1, those with CD4+/CD8+ T-cell ratio≥1 had significantly higher median %CD8 naive T-cells (25.4 (14.0–36.0)% versus 14.4(9.4–21.6)%, p<0.0001), but significantly lower absolute CD8+ T-cell count (464(384.5-567) versus 765 (603-1084) cells/mm^3^, p<0.001). This observation persisted when the analysis was restricted to those on ART with suppressed HIV RNA.

### Usefulness of CD4+/CD8+ T-cell ratio, CD4+ and CD8+ in informing on expanded T-cell subsets

**Figure 1:**
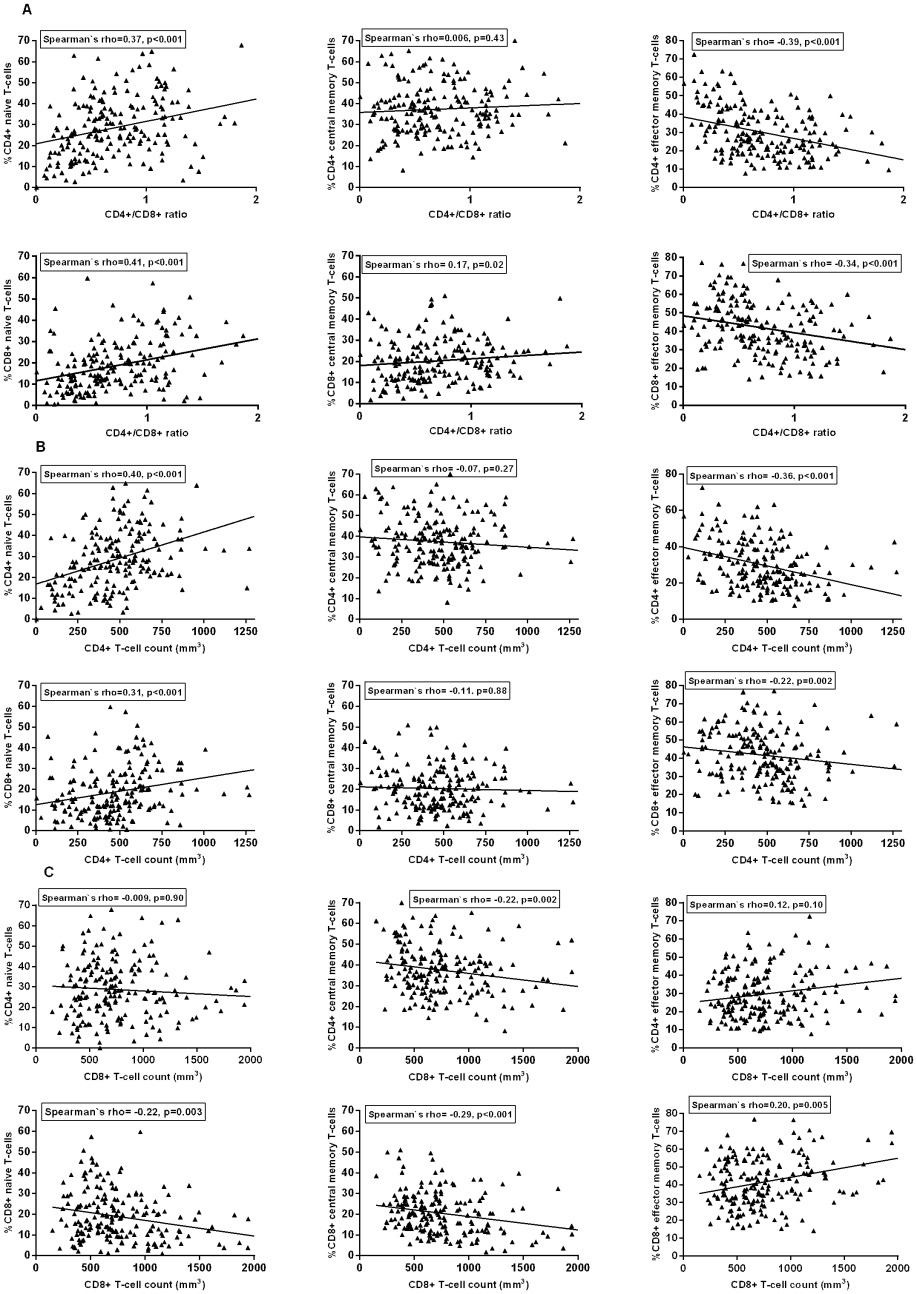
Correlation between CD4+ and CD8+ expanded T-cell subsets with CD4+/CD8, CD4+ and CD8+ T-cells.

We further explored whether as a surrogate marker of immune response, the CD4+/CD8+ T-cell ratio is a better indicator of expanded T-cell subset, compared to CD4+ and CD8+ T-cell count. In the unadjusted analysis, the CD4+/CD8+ T-cell ratio showed the strongest association to %CD4+ effector memory T-cells (rho = −0.39, p<0.001), %CD8+ naive T-cells (rho = +0.41, p<0.001) and %CD8 effector memory T-cells (rho = −0.34, p<0.001), compared to CD4+ and CD8+ T-cell count (Figure 1). CD4+ T-cell count showed the strongest association to %CD4+ naïve T-cells (rho = +0.40, p<0.001) compared to the CD4+/CD8+ T-cell ratio and CD8+ T-cell count. CD8+ T-cell count showed the strongest association to %CD4+ central memory T-cells (rho = −0.22, p = 0.002) and %CD8 central memory T-cells (rho = −0.29, p<0.001). These crude associations rather suggest that CD4+/CD8+ T-cell ratio could be more informative on %CD4+ effector memory, %CD8+ naive and %CD8+ effector memory T-cells, while CD4+ T-cell count could be more informative on %CD4 naïve T-cells and CD8+ T-cell count on %CD8 central memory T-cells and %CD4+ central memory T-cells.

In the multivariable analysis, adjusted for demographic and treatment-related variables most likely to exert confounding effect, a series of linear regression models with either CD4+/CD8+ T-cell ratio, CD4+ or CD8+ T-cell count as an independent variable was fitted for each expanded T-cell subset. Based on fit indices, AIC and BIC, %CD8+ naïve, %CD4 naïve, %CD8+ effector memory and %CD4+ effector memory T-cells were better explained by a model with CD4+/CD8+ T-cell ratio as an independent variable (Table S1). %CD4+ and %CD8+ central memory T-cells were better explained by a model with CD8+ T-cell count as an independent variable.

## Discussion

To our knowledge this is the first large study in a diverse cohort of adults patients to explore the role of expanded CD4+ and CD8+ T-cell lymphocytes subsets on CD4+/CD8+ T-cell ratio normalization and the usefulness of CD4+/CD8+ T-cell ratio, CD4+ and CD8+ T-cell count in informing on the expanded T-cell subsets, with analyses correcting for all relevant clinical covariates. The principal finding of this study is the observation that, after adjustment for factors commonly reported as associated with ART-induced immune responses, those patients with a higher percentage of naïve CD8+ T-cells are more likely to attain normalization of their CD4+/CD8+ T-cell ratio. Relationship between CD4+/CD8+ T-cell ratio and expanded T-cell subsets have previously been described in a small cohort of children and adolescents [21] and recently in 47 ART-naïve HIV-infected individuals [22]. Our study further demonstrates that, the CD4+/CD8+ T-cell ratio might be used to inform on CD4+ and CD8+ naïve and effector memory T-cell subsets while CD8+ T-cell count might be used to inform on CD4+ and CD8+ central memory T-cells.

We chose a higher CD4+/CD8+ T-cell ratio as a reflection of a more ‘normalized’ immune response. Although the CD4+ T-cell count remains a robust predictor of mortality, both in those on and not on ART [23], immune responses to antiretroviral treatment of HIV infection are varied and involve changes in both CD4+ and CD8+ T-cell repertoires, both of which are incorporated into the CD4+/CD8+ T-cell ratio. Although few studies have examined the relationship between CD4+/CD8+ T-cell ratio and clinical outcomes, the CD4+/CD8+ T-cell ratio is inverted (<1) in HIV infection, falls as HIV disease progresses and in most cases increases with exposure to ART. Although recent studies have shown independent associations between lower CD4+/CD8+ T-cell ratio, subclinical markers of age related diseases [24], risk of non-AIDS morbidity or mortality [25] and persistent immune activation despite ART [15], a large Canadian cohort study failed to find an association between normalization of the CD4+/CD8+ ratio (defined as a ratio ≥1.2) and the probability of AIDS defining illness or death [14]. Despite the lack of clear association with clinical outcomes, we considered CD4+/CD8+ T-cell ratio to be a relatively simple and robust endpoint for examination of immune responses in HIV.

In this cohort with a majority of the subjects virologically suppressed on effective ART, we observed that higher percentage of naïve CD8+ T-cells was associated with higher CD4+/CD8+ T-cell ratio, with a significantly higher naïve CD8+ T-cell percentage in those with a CD4+/CD8+ T-cell ratio >1, while higher percentage of effector CD4+ T-cells were associated with lower CD4+/CD8+ T-cell ratio in adjusted analyses. Those with normalized CD4+/CD8+ T-cell ratio were also shown to have lower absolute CD8+ T-cell counts, indicative of a less activated immune system, consistent with previous studies [15].

With most patients on ART being able to attain virological suppression, there has been a shift in focus to long-term treatment outcomes impacted by emerging long-term co-morbidities such as cancers, cardiovascular disease, low bone mineral density and other age–related illnesses, commonly referred to as non-AIDS illnesses, which account for a significant proportion of mortality in those on ART [23]. Persistent immune activation despite effective ART has been implicated in the pathogenesis of these co-morbidities [26], [27]. Although we did not determine markers of T-cell activation, our findings are consistent with previous studies showing associations between lower CD4+/CD8+ T-cell ratio and both T-cell and B-cell activation [15] and support an important role for naïve CD8+ T-cells in maintaining a less activated immune system, reflected in the association between higher naïve CD8+ T-cell percentage and both lower CD8+ T-cell count and higher CD4+/CD8+ T-cell ratio. These data are consistent with a previous study in untreated patients with HIV in which the naïve CD8+ T-cell pool was lost over time with higher naïve CD8+ T-cells associated with lower absolute CD8+ T-cell counts [28], although relationships between these factors and the CD4+/CD8+ T-cell ratio were not examined. We have shown that, even in patients on effective ART with viral suppression, the inverse relationship between naïve CD8+ T-cell percentage and absolute CD8+ T-cell count persists and moreover, we have related it to elevated CD4+/CD8+ T-cell ratio. Based on this, naïve CD8+ T-cells may provide a useful marker to distinguish individuals who are very different immunologically, but whether or not measurement of naïve CD8+ T-cells predicts those who may have better immunological responses to ART remains to be seen.

Our findings are also consistent with previous studies highlighting a role for naïve CD8+ T-cells in controlling immune responses to infection. In a mouse model of sepsis, early loss of naïve CD8+ T-cells is associated with subsequent delayed recovery of primary CD8+ T-cell responses, which is thought to predispose to higher risk of infections from intracellular pathogens such as viruses during the post-sepsis recovery period [29]. Indeed, the naïve CD8+ T-cell pool is important in directing the primary host immune responses [30]. In animal models, an enhanced naïve CD8+ T-cell pool is associated with better immune responses to viral infection, with availability of a more balanced repertoire of viral-specific epitopes available in the naïve CD8+ T-cell pool compared to the memory CD8+ T-cell pool [31]. Our findings of an association between a higher naïve CD8+ T-cell percentage and a more normal immune phenotype as suggested by a higher CD4+/CD8+ ratio is consistent with an immune system that is capable of greater recovery from chronic viral infection with antiretroviral therapy. Whether pre-ART naïve CD8+ T-cell percentages predict immune recovery and defining the role of naïve CD8+ T-cells in the individual response to other strategies that rely on host immune responses to control viral infection, such as that proposed with HIV cure [32] and therapeutic vaccination strategies are worthy of further research, particularly in determining the optimum time for ART initiation to preserve both CD4+ and CD8+ T-cell function.

Observed associations between CD4+/CD8+ T-cell ratio and nadir CD4+ T-cell count, cumulative exposure to ART and absolute CD8+ T-cell count are consistent with findings from univariate analyses in a previous study and a large cohort study [14] and reinforce the importance of continued effective ART in driving evolution of immune responses to HIV infection.

Previously, in a group of ART-naive HIV-infected patients, compared to CD4+ T-cell count, the CD4+/CD8+ T-cell ratio was shown to be a better predictor of “combined” T-cell pathogenesis that occurs in HIV infection [22]. While that particular study combined different markers of T-cell pathogenesis, we have shown that specifically for individual naïve and memory T-cell subsets, the CD4+/CD8+ T-cell ratio might be more informative on CD4+ and CD8+ naïve and effector memory T-cells compared to the CD4+ and CD8+ T-cell count.

Our study is subject to a series of limitations. Firstly, although the analysis was adjusted for major factors that have been shown to affect CD4+/CD8+ T-cell ratio such as age, gender, ethnicity, Hepatitis C and HIV RNA levels, some individuals may be genetically pre-disposed to high or low CD4+/CD8+ T-cell ratio [33]. Secondly, while this exploratory analysis suggests an important role of naïve CD8+ T-cell and effector CD4+ T-cell subsets in immunological response, we were limited in the number of cell surface markers we could measure and did not include markers of T-cell activation. Lastly, the cross-sectional nature of the study prevents us from determining definitive cause/effect relationships, with prospective studies required to determine the role of naïve CD8+ T-cells in predicting normalization of CD4+/CD8+ T-cell ratio.

Nevertheless, this study provides important and new insights into the potential role of CD4+ and CD8+ T-cell subpopulations in immunological responses in HIV and in particular suggests an important role for naïve CD8+ T-cells in normalisation of the immune response in a large cohort of subjects with the majority receiving effective ART. If naïve CD8+ T-cells do predict a better immune response, these data suggest that a more detailed examination of immune responses may be useful within routine clinical practice, particularly in those with HIV on ART.

## Supporting Information

Figure S1 **Gating strategy used to discriminate CD4+ and CD8+ T-cell subsets.** Note: In the dot Plot of CD62L PE versus CD45RO APC, Q1 displays CD62L+CD45RO-cells (naïve cells). Q2 displays CD62L+CD45RO+ cells (central memory cells), Q3 displays CD62L-CD45RO- cells (revertant memory cells), Q4 displays CD62L-CD45RO+ cells (effector memory cells).
(TIF)

Table S1 **Fit indices for expanded T-cell subset models with CD4+/CD8+ T-cell ratio, CD4+ or CD8+ T-cell count.**
(DOCX)
