# Supplementary figures and images for: Clinical, Immunological and Treatment-Related Factors Associated with Normalised CD4+/CD8+ T-Cell Ratio: Effect of Naïve and Memory T-Cell Subsets
Source: PLoS One. 2014 May 9;9(5):e97011. doi: 10.1371/journal.pone.0097011 (PMC4016205; doi:10.1371/journal.pone.0097011)

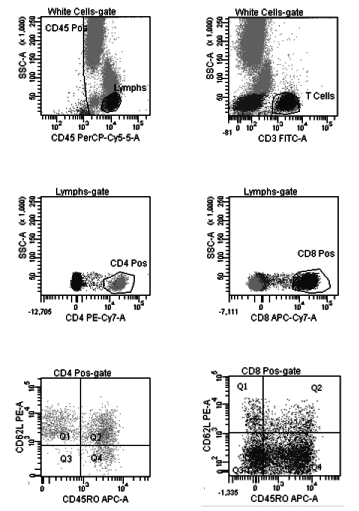

Supplement: Figure S1 — Gating strategy used to discriminate CD4+ and CD8+ T-cell subsets. Note: In the dot Plot of CD62L PE versus CD45RO APC, Q1 displays CD62L+CD45RO-cells (naïve cells). Q2 displays CD62L+CD45RO+ cells (central memory cells), Q3 displays CD62L-CD45RO- cells (revertant memory cells), Q4 displays CD62L-CD45RO+ cells (effector memory cells). (TIF) [file pone.0097011.s001.tif]
