# Supplementary material for: Clinical, Immunological and Treatment-Related Factors Associated with Normalised CD4+/CD8+ T-Cell Ratio: Effect of Naïve and Memory T-Cell Subsets
Source: PLoS One. 2014 May 9;9(5):e97011. doi: 10.1371/journal.pone.0097011 (PMC4016205; doi:10.1371/journal.pone.0097011)
Supplement: Table S1 — Fit indices for expanded T-cell subset models with CD4+/CD8+ T-cell ratio, CD4+ or CD8+ T-cell count. (DOCX) [file pone.0097011.s002.docx]

Table S 1: Fit indices for expanded T-cell subset models with CD4+/CD8+ T-cell ratio, CD4+ or CD8+ T-cell count.

|  | *%CD4+ naïve models | | *%CD4+ effector memory models | | *%CD4+ central memory models | | *%CD8+ naïve models | | *%CD8+ effector memory models | | *%CD8+ central memory models | |
| --- | --- | --- | --- | --- | --- | --- | --- | --- | --- | --- | --- | --- |
|  | AIC | BIC | AIC | BIC | AIC | BIC | AIC | BIC | AIC | BIC | AIC | BIC |
| CD4+/CD8+ T-cell ratio | **1487.4** | **1516.4** | **1470.6** | **1499.7** | 1445.8 | 1474.9 | **1392.8** | **1421.9** | **1476.4** | **1505.5** | 1367.7 | 1396.8 |
| CD4+ T-cell count | 1491.7 | 1520.8 | 1486.2 | 1515.3 | 1452.6 | 1481.6 | 1416.8 | 1445.9 | 1497.6 | 1526.7 | 1376.5 | 1405.6 |
| CD8+ T-cell count | 1502.6 | 1531.7 | 1484.4 | 1513.5 | **1439.6** | **1468.7** | 1416.8 | 1445.9 | 1488.8 | 1517.9 | **1361.5** | **1390.6** |

Best fitting model is in bold; AIC= Aikike Information Criterion; BIC=Bayesian Information Criterion

*All models adjusted for age, gender, ethnicity, Hepatitis C Ab status HIV RNA, and cumulative exposure years to ART
